# Livestock-associated methicillin-resistant *Staphylococcus aureus* along the pork food chain: a study of four commercial pig fattening farms in north-east Germany and survival in processed meat products

**DOI:** 10.3389/fmicb.2026.1928317

**Published:** 2026-10-27

**Authors:** Marco Ebert, Sandra Rose, Michael Biggel, Alexandra Fetsch

**Affiliations:** 1 Department of Agriculture and Food Sciences, Neubrandenburg University of Applied Sciences (HS NB), Neubrandenburg, Germany; 2 Faculty of Agriculture, Civil and Environmental Engineering, University of Rostock, Rostock, Germany; 3 Institute for Food Safety and Hygiene, University of Zurich (UZH), Zurich, Switzerland; 4 Competence Centre for Food Safety, Chair of Food Microbiology, Faculty of Veterinary Medicine, Ludwig-Maximilians-Universität München (LMU Munich), Munich, Germany; 5 Department Biological Safety, German Federal Institute for Risk Assessment (BfR), Berlin, Germany

**Keywords:** antimicrobial resistance, farm-to-fork, fattening pigs, food safety, LA-MRSA, meat products, ST398, whole-genome sequencing (WGS)

## Abstract

**Introduction:**

Livestock-associated methicillin-resistant *Staphylococcus aureus* (LA-MRSA) of Sequence Type (ST) 398 is widespread in European pig farming, yet molecular evidence linking specific farm strains to finished pork products remains limited.

**Methods:**

Four commercial pig fattening farms in northeastern Germany were sampled for MRSA across the full pork production chain, from nasal swabs collected from pigs and environmental samples taken on farms, through slaughter, to four processed meat products representing a gradient of processing intensity (*Hackepeter*, *Frische Zwiebelmettwurst*, *cervelat-type sliceable raw sausage*, and *Lyoner*). MRSA isolation followed two-step selective enrichment culture with confirmation by multiplex Real-Time PCR; all isolates were characterized by *spa*- and SCC*mec*-typing and subjected to whole-genome sequencing with Core Genome Multilocus Sequence Typing (cgMLST)-based cluster analysis using a 1,861-locus *Staphylococcus aureus* scheme.

**Results:**

MRSA was detected on 3/4 farms (75%); all pooled nasal swab samples from positive farms were positive at both the farm (7/7 pools) and slaughterhouse (8/8 pools) levels. cgMLST revealed that pest flies carried ST398 strains genomically nearly identical (as few as 1 allele) to those colonizing farm pigs, identifying farm-associated pests as potential carriers of LA-MRSA. Among finished products, MRSA was not detected in seasoned raw minced meat (*Hackepeter*; 0/9) and heat-treated cooked sausage (*Lyoner*; 0/12). In contrast, LA-MRSA ST398 (t034/SCC*mec* V) survived in spreadable raw sausage (*Frische Zwiebelmettwurst*; 6/12 positive) through the full seven-day shelf life; a single ST398 MRSA (t011/SCC*mec* V) was detected in *cervelat-type sliceable raw sausage* after fermentation.

**Discussion:**

Whole-genome sequencing and cgMLST confirmed the recent common ancestry of ST398 isolates recovered from pig nasal swabs, farm environments, slaughter stages, and final meat products, including raw fermented sausages, providing genomic evidence of LA-MRSA transmission along the farm-to-fork chain. Human-associated MRSA (ST39, t007/SCC*mec* II) was detected exclusively at the slaughterhouse and the meat processing stage and carried the human-associated immune evasion and *egc* enterotoxin gene clusters, absent from all ST398 isolates, implicating that the slaughterhouse may be an entry point for a non-livestock MRSA lineage into the pork food chain.

## 1 Introduction

Methicillin-resistant *Staphylococcus aureus* (MRSA) of Sequence Type (ST)398, primarily represented by *spa*-types t034 and t011 with SCC*mec* type V, constitutes the predominant livestock-associated MRSA (LA-MRSA) lineage in Europe (Fetsch and Johler, 2026), and is a common pig-associated problem worldwide. The prevalence of LA-MRSA in pigs is extremely heterogeneous across geographical region, herd structure, and sampling method, with prevalence rates of up to 89% coming from parts of Europe (Fetsch et al., 2021). The ST398 lineage has proved remarkably persistent: despite a 70% reduction in veterinary antimicrobial use in the Netherlands since 2009, approximately 76% of Dutch pig farms remain MRSA-positive (Dierikx et al., 2016). This observation likely reflects ecological adaptation, within-farm transmission, and reintroduction via animal movement rather than simple antibiotic selection pressure (van Duijkeren et al., 2025).

LA-MRSA, particularly CC398, represents an important occupational and zoonotic health hazard, with livestock exposure strongly associated with human colonization and potential onward transmission (Bisdorff et al., 2012; Kinross et al., 2017). Farm air, dust and other environmental matrices may additionally act as reservoirs and transmission pathways, although their contribution to the overall human health burden remains insufficiently quantified (Bos et al., 2016; EFSA Panel on Animal Health and Welfare et al., 2022). LA-MRSA can also spread along the food production continuum through several key transmission points. Colonization levels differ considerably among livestock species, with pigs showing the highest prevalence. For instance, in the Netherlands, LA-MRSA was detected on 76% of pig farms, compared with 25% of veal calves, 4.5% of sheep, and 0% of broiler farms (van Duijkeren et al., 2025). Environmental dissemination may occur via airborne particles emitted from livestock facilities, although transmission appears to be restricted to relatively short distances (Angen et al., 2021). Slaughter and processing environments constitute important hubs for bacterial spread, facilitating the transfer of MRSA between animals, equipment, meat products, and workers. The detection of closely related MRSA ST398 strains across these compartments demonstrates the capacity of the pathogen to persist throughout the production chain, highlighting the need for effective hygiene and biosecurity measures during slaughter and processing (Bouchami et al., 2020).

Globally, MRSA has also been detected in a wide range of meat and meat products. A recent systematic review and meta-analysis of 34,103 samples from 98 studies quantified the pooled MRSA prevalence at 3.72% across all meat categories, with processed meat products at 2.84% and cooked meat effectively at zero (Xing et al., 2025). European estimates are broadly consistent with these figures; in raw livestock meat specifically, pooled prevalence approaches 3.86% (Xing et al., 2025). Recent studies indicate the emergence of new food-associated MRSA lineages with enhanced virulence potential. In China, ST7-t091 has become a dominant genotype in pork and frequently carries a broad repertoire of enterotoxin genes, including the recently described *selw* (Wang et al., 2021). In addition, poultry products have been identified as a potential reservoir of ST398/ST9 MRSA strains closely related to isolates recovered from human infections (Fetsch et al., 2017). These developments underline the importance of integrated surveillance and detailed molecular characterization of MRSA throughout the food production chain (Fetsch and Johler, 2026). Particularly, the consumption of raw or minimally processed pork products, which are traditional items in German cuisine, represents a potential exposure route that warrants investigation.

Despite the substantial proportion of MRSA-positive pig fattening operations, the extent to which specific strains found in live animals can be traced all the way to the consumer, through slaughter and into processed meat products, remains poorly understood. This study therefore aimed to (i) trace MRSA from pig farms through slaughter to processed meat products using whole-genome sequencing (WGS), and (ii) determine MRSA survival across four meat products spanning a gradient of processing intensities.

## 2 Materials and methods

### 2.1 Study design and farms

Four commercial pig fattening farms in the Ludwigslust-Parchim district (Mecklenburg-Western Pomerania, Germany) were sampled (Table 1). We designed the study as a targeted farm-to-fork traceability investigation, prioritizing complete pig-to-product continuity over statistical representativeness of German pig farms; farms were accordingly selected for their capacity to support this traceability rather than through random or stratified sampling. Farm 1 operated in a closed production system; the others did not. Nasal swabs from up to nine animals per bay were pooled; individual traceability was maintained via ear tags and ham-stamp numbering applied before slaughter. Pool sizes and the resulting number of pools varied between farms because the number of pigs available within individual pens and slaughter batches differed according to herd structure and slaughter logistics. Animals from MRSA-positive farms were re-sampled at the slaughterhouse; their meat was then used to manufacture four product types at Neubrandenburg University of Applied Sciences (HS NB). All MRSA isolates were characterized and subjected to WGS.

**Table 1:** Characteristics of the four pig fattening farms included in the study.

Farm	Number of finishing places	Number of pigs/bay	Slaughter weight of pigs (kg)	Pigs sampled (*n*)	Pooled samples (*n*)
1	350	15–18	118	9	1
2	350	15–20	150	6	2
3	1,500	10–12	118	9	3
4	4,500	10	118	9	3

Sampling was conducted as follows for farm 1: 9 nasal swabs in 1 pooled sample; for farm 2: 6 nasal swabs in 2 pooled samples; for farm 3 & 4: 9 nasal swabs in 3 pooled samples. Additionally, one environmental sock swab per farm and pests (a fly at farm 1, a fly and a spider at farm 2, a fly and a mouse at farm 3 and a fly at farm 4) were collected.

### 2.2 Sampling

For an overview of the sampling activities within the project, see Figure 1. At each farm, pooled nasal swabs (sterile viscose-tip swabs in Amies gel medium; 11,770 M, Süsse Labortechnik, Gudensberg, Germany) and environmental sock swabs (one per farm) were collected in the year 2016. Sampling was deliberately restricted to a single period (May–July) to minimize temporal variability between farms. The study was exempt from ethics approval, as samples were collected from animals at farms and commercial slaughter in the context of routine food-safety activities. Dead pests (flies, a mouse, and a spider) were individually bagged (*n* = 6). To minimize the risk of cross-contamination between farms, sampling was performed under strict biosecurity conditions. The sampler had no contact with other pig farms for at least 48 h before each farm visit and used sterile sampling equipment and appropriate aseptic techniques throughout sample collection. Animals from the MRSA-positive farms were sampled again on their respective slaughter days: post-mortem for farm 1 and ante-mortem for farms 3 and 4. Since two animals from farm 4 were not slaughtered on the same day as the rest of the group, only seven animals from farm 4 were sampled at slaughter. In contrast, all nine animals from farms 1 and 3 were examined at slaughterhouse-level. Slaughterhouse environmental samples comprised quarantine-pen sock swabs (before and after animal housing), scalding water, and surface swabs (∼20 cm^2^) from the band saw blade and cutting knife (one per farm). All samples were transported to the laboratory of the HS NB on the same day as collection, at a maximum temperature of 18 °C. After chilling overnight at 7 °C, meat fractions (lean cuts, back fat, belly meat, and cheek meat) were transported to the HS NB, sorted by farm, minced through a 3-mm perforated disc, and tested for MRSA (four samples per farm; *n* = 12). Minced meat was vacuum-packed and frozen at −18 °C.

**Figure 1:**
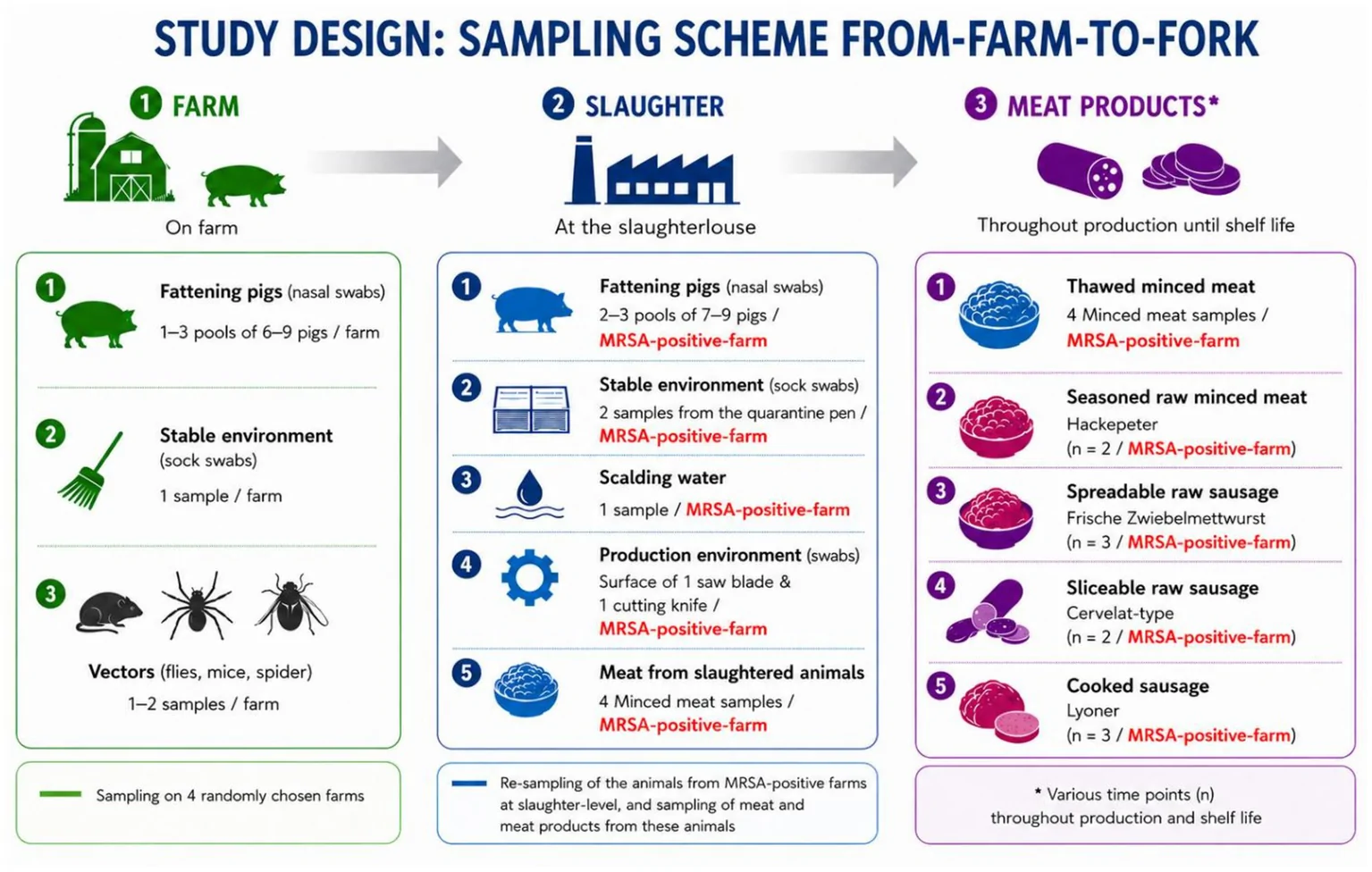
Sampling scheme from farm-to-fork followed in the project. The graphic was designed by the authors and created with the help of ChatGPT (ChatGPT Go Modell GPT-5.5 Instant, OpenAl, 2026).

After approximately 2 months at −18 °C, the minced meat was thawed at 2 °C, tested again for MRSA and processed into four product types representing a gradient of processing intensity: (i) *Hackepeter* (seasoned raw minced meat; no preservation steps; sampled, after mixing, and after 24 h); (ii) *Frische Zwiebelmettwurst* (spreadable raw sausage; fermented at 7 °C for 48 h; sampled after mixing of ingredients, after 50 h of fermentation, and after 7 days/end of shelf life); (iii) *cervelat-type sliceable raw sausage* (fermented at 20 °C for 96 h in a climate chamber; sampled after mixing, and at the end of fermentation); and (iv) *Lyoner* (cooked sausage; heat-treated at 75 °C for 45 min; sampled after mixing, after heating, and after 7 days at 7 °C). Full recipes, pH and a_W_ (water activity) value measurement results are provided in Supplementary Materials S1: Tables S1-1–S1-9.

Project staff (*n* = 3), taking samples and preparing meat products, were screened for MRSA by nasal swab at project start and completion.

### 2.3 Microbiological analysis

MRSA isolation followed a two-step selective enrichment as previously described (Fetsch et al., 2016). In brief, 24 h of pre-enrichment in Mueller-Hinton Broth supplemented with 6% NaCl (37 °C) was followed by transfer to CASO broth containing cefoxitin (3.5 mg/L) and aztreonam (50 mg/L) for 24 h, followed by plating onto ChromID™ MRSA agar (bioMérieux, Nürtingen, Germany). Isolates were send to the BfR and screened as MRSA by means of MALDI-TOF mass spectrometry and confirmed by applying an in-house multiplex-real-time PCR adapted from Kilic et al. (2010) and Fosheim et al. (2011). SCC*mec*- and *spa*-typing were also conducted as previously described (Kraushaar and Fetsch, 2014).

### 2.4 Whole-genome sequencing and bioinformatic analysis

Isolates were stored at −80 °C and subjected to short-read WGS by Novogene (Munich Omics Centre, Germany), commissioned by Ludwig-Maximilians-Universität München (Munich, Germany) in 2026. The Qiagen DNeasy Blood & Tissue Kit was used for DNA extraction. Sequencing libraries were prepared at Novogene (Munich Omics Centre, Germany) using the Novogene NGS DNA Library Prep Set (Cat. No. PT004). The library was quantified through Qubit and qPCR, and size distribution detected with fragment analyzer. Quantified libraries were pooled and sequenced on the NovaSeq X Plus platform using the PE150 configuration. Illumina read adapters and low-quality bases were trimmed with fastp (Chen, 2023), and draft genomes were assembled using SPAdes 3.14.1 (Prjibelski et al., 2020) implemented in shovill 1.1.0. Assembly quality was assessed using seqkit 2.13.0 (Shen et al., 2024) and CheckM2 1.1.0 (Chklovski et al., 2023). The per-isolate quality metrics (Q30, N50, scaffold count, and coverage) are detailed in Supplementary Material S2; Table S2-1. Sequence types were determined using PubMLST (Jolley and Maiden, 2010). Core-genome MLST (cgMLST) distances were calculated using pyMLST 2.3.1 (parameters -c 0.9, −i 0.9) (Biguenet et al., 2023) with the 1,861-locus *Staphylococcus aureus* (*S. aureus*) scheme from cgmlst.org. The software PHYLOViZ Online was used for figure creation based on the analysis of the allelic profiles and their associated epidemiological data. AMRFinderPlus 4.2.5 (database version 2025-12-03.1) was used for the detection of resistance genes (Feldgarden et al., 2021), and ABRicate 1.0.1^1^ (minimum coverage, 70%; identity, 70%) in combination with VFDB set B was used for the detection of virulence genes (Zhou et al., 2025).

### 2.5 Statistical analysis

Prevalence and survival data are reported descriptively because of the small number of farms/clusters and non-independence of repeated within-farm sampling; Differences in virulence and AMR gene carriage between the ST398 and ST39 lineages were assessed using Fisher’s exact test (two-tailed); given the small, pre-specified panel of lineage-differentiating genes identified from the cgMLST/phylogenetic grouping (rather than an unbiased genome-wide screen), no correction for multiple comparisons was applied.

## 3 Results

An overview of MRSA detection across all sampling stages and farms is provided in Table 2. For detailed results, see Supplementary material Table S3.

**Table 2:** MRSA detection by sample type, food-chain stage, and farm.

Sample type	Stage/Meat category	Farm 1	Farm 3	Farm 4	Total
Nasal swabs (pooled)	Farm	1/1	3/3	3/3	7/7
Sock swabs	Farm	0/1	0/1	0/1	0/3
Pests	Farm	0/1	2/2	1/1	3/4
Nasal swabs (pooled)	Slaughterhouse	3/3*	3/3**	2/2**	8/8
Quarantine sock swabs	Slaughterhouse	0/2	0/2	0/2	0/6
Scalding water	Slaughterhouse	0/1	0/1	0/1	0/3
Band saw blade	Slaughterhouse	1/1	0/1	0/1	1/3
Cutting knife	Slaughterhouse	0/1	1/1	0/1	1/3
Minced meat, pre-freeze	Pre-processed meat	3/4	2/4	1/4	6/12
Minced meat, post-thaw	Pre-processed meat	0/4	1/4	1/4	2/12
Hackepeter	Meat product	0/2	0/2	0/2	0/6
Frische Zwiebelmettwurst	Meat product	0/3	2/3	2/3	4/9
Cervelat-type sausage	Meat product	0/2	0/2	1/2	1/6
Lyoner (cooked)	Meat product	0/3	0/3	0/3	0/9

Values show MRSA-positive samples/total tested. Farm 2 (MRSA-negative) was excluded after farm-level screening.

^*^Sampling post-mortem.

^**^Sampling ante-mortem.

### 3.1 Sampling and detection rates

MRSA was detected on 3/4 farms (75%; farms 1, 3, and 4). Farm 2 was negative in all nasal swabs and environmental samples and was excluded from further sampling.

All pooled nasal swabs from positive farms were positive at both the farm level (7/7 pools) and the slaughterhouse level (8/8 pools). Sock swabs were negative at all farms (0/4) and slaughterhouse quarantine-pen (0/6) sampling points, as were all scalding water samples (0/3). Among pest samples, MRSA was detected in two flies (farms 3 and 4), and a mouse (farm 3); no MRSA was detected in the fly sampled at farm 1 (3/4 samples from MRSA-positive farms, 75%) or in a fly and a spider sampled at farm 2.

In pre-processed minced meat, MRSA was detected pre-freeze (6/12) and post-thaw (2/12). In the final meat products, MRSA was detected in *Frische Zwiebelmettwurst* (6/12), and *cervelat-type sliceable raw sausage* (1/9), but neither in *Lyoner* nor *Hackepeter*.

All nasal swabs taken from the project staff were MRSA-negative at the beginning and end of the project.

### 3.2 Molecular typing and WGS-based traceability

All 34 strains (33 MRSA and one *mecA*-negative *S. aureus)* recovered along the pork production chain belonged to three distinct STs (Table 3). The dominant lineage comprised 26 strains of ST398, the LA-MRSA lineage prevalent in European pig production. A second cluster consisted of seven strains of ST39 (*spa* type t007, SCC*mec* type II) detected exclusively at the slaughter and food processing stages. A single strain of ST15 (*spa* type t346) was recovered from a slaughterhouse cutting knife, that had been used to cut up carcasses from farm 4, and was confirmed as a *mecA*-negative *S. aureus*.

**Table 3:** Strain characterisation summary of 33 MRSA isolates including *spa*-type, SCC*mec* type, and MLST type.

*spa*-type	SCC*mec* type	MLST	*n* (% of 33 MRSA*)
t034	V	ST398	21 (64%)
t007	II	ST39	7 (21%)
t011	V	ST398	3 (9%)
t2011	V	ST398	1 (3%)
t1255	V	ST398	1 (3%)

^*^A total of 34 strains were isolated in the project (33 MRSA and one mecA-negative *S. aureus*). The mecA negative *S. aureus* (BfR-ST-02950) was characterised as spa-type t346 and MLST type ST15.

The minimum spanning tree (MST) constructed from cgMLST allelic profiles in PHYLOViZ Online illustrates the genetic structure of the collection: a large, interconnected ST398 clade forms the central topology, while the ST39 cluster and the ST15 isolate each represent distant outlier lineages, separated from the ST398 hub by approximately 1,645 and 1,739 allelic differences, respectively (Figure 2).

**Figure 2:**
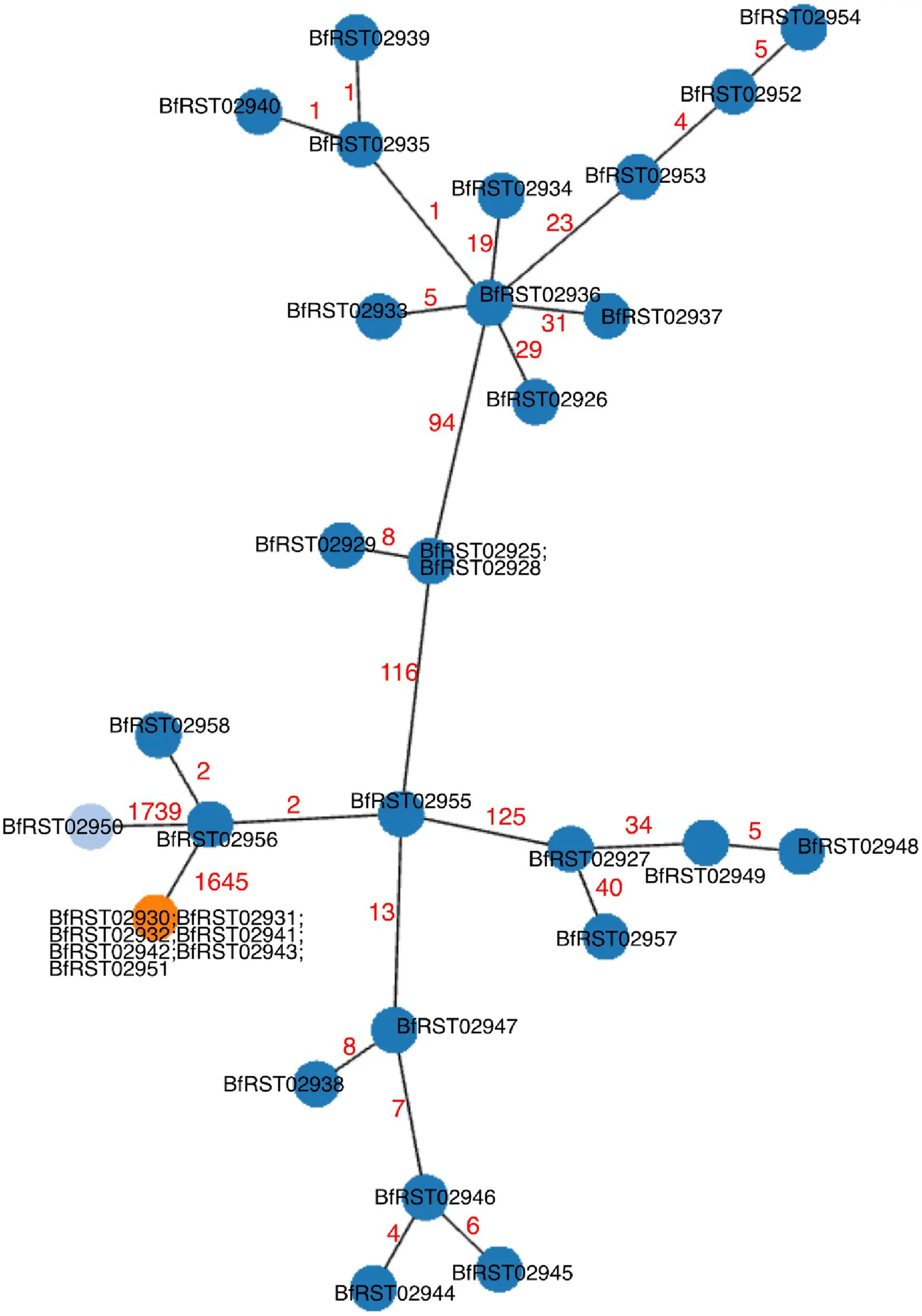
Minimum spanning tree (MST) of cgMLST allelic profiles of MRSA (*n* = 33) and *S. aureus* (*n* = 1) strains recovered along the pork production chain. The MST was constructed using PHYLOViz Online based on core genome multilocus sequence typing (cgMLST) allelic profiles of 34 strains (BfRST02925–BfRST02958) isolated from three pig farms (Farms 1, 3, 4) and associated slaughterhouses and food products. Each node represents one cgMLST allelic profile. Coloring of nodes indicates ST398 cluster (dark blue), ST39 cluster (orange), and ST15 single strain (light blue), respectively. Red numbers between nodes indicate allelic differences.

In the following, we describe the findings within the three distinct genomic clusters in more detail. Respective allelic profiles and the distance matrix are provided in Supplementary Material S2 (Tables S2-2 and S2-3).

#### 3.2.1 ST398 clade

Multiple transmission clusters (≤8 alleles) were detected within the ST398 clade. Strains recovered from different pigs on farm 4 (BfR-ST-02944, BfR-ST-02945, BfR-ST-02946) were separated by only four and six allelic distances. Likewise, strains from different pigs in farm 3 (BfR-ST-02933, BfR-ST-02935) differed by 6 allelic distances only, suggesting the circulation of closely related strains within the herd. Farm-to-slaughterhouse continuity was demonstrated in two farms: (i) farm 3, where nasal swab isolates from pigs sampled at the slaughterhouse (BfR-ST-02939 and BfR-ST-02940) differed by only one allele from an on-farm nasal swab isolate (BfR-ST-02935); (ii) farm 1, where a strain isolated at farm level (BfR-ST-02925) was almost identical (8 allelic differences) to another isolate from slaughter (BfR-ST-02929). These findings indicate that the same strains colonizing pigs on the farm were detectable in the same herd at the point of slaughter. Isolates (BfR-ST-02948, BfR-ST-02949) recovered at slaughter from different animals originating from the same farm (farm 4) also showed high genetic homogeneity, with five allelic distances only. The most prominent evidence of recent common ancestry of MRSA from farm to the final product was identified among isolates with farm 4 origin. Here, an MRSA isolated at farm level from a pest fly (BfR-ST-02947) differed by 13 and 15 allelic distances from two pre-processed meat samples (BfR-ST-02955 and BfR-ST-02956), respectively.

Environmental sampling at farm level identified pest animals as carriers of ST398 strains closely related to those colonizing farm pigs. At farm 3, a pest fly (BfR-ST-02936) yielded a MRSA isolate clustering within the farm 3 ST398 sub-clade at allelic distances of five (BfR-ST-02933) and one (BfR-ST-02935) allele from adjacent pig nasal swab isolates, respectively. At farm 4, a pest fly (BfR-ST-02947) clustered within the farm 4 subclade at close allelic distance (7 alleles) from on-farm (BfR-ST-02944, BfR-ST-02945) and at-slaughter (BfR-ST-02946) pig isolates.

At slaughter level, the MST topology revealed additional evidence of genomic homogeneity between strains. BfR-ST-02938, a nasal swab isolate from farm 3 pigs sampled at slaughter, was positioned within the farm 4 right-branch sub-clade of strains originating from animals (BfR-ST-02944 - BfR-ST-02946) and the environment (BfR-ST-02947) at farm 4. The distance between an MRSA isolated from the band saw blade (BfR-ST-02929) during slaughtering of farm 1 animals and the on-farm isolate (BfR-ST-02925) was eight alleles, only.

Looking at the processing and final product level, two strains derived from the pre-processed meat (BfR-ST-02955, BfR-ST-02956) and one strain (BfR-ST-02958) isolated from the final meat product (*Frische Zwiebelmettwurst*) prepared from animals raised at farm 4 were separated by only two allelic distances each, indicating near-identical cgMLST profiles. Similarly, isolates from pre-processed meat (BfR-ST-02952, BfR-ST-02953) and the final meat product (*Frische Zwiebelmettwurst*, BfR-ST-02954) showed allelic distances of no more than five and seven, respectively.

#### 3.2.2 ST39 cluster

Seven strains belonging to ST39 (BfR-ST-02930 to BfR-ST-02932, BfR-ST-02941 to BfR-ST-02943, BfR-ST-02951) shared *spa* type t007, SCC*mec* type II, and identical cgMLST profiles. This cluster was detected exclusively at the slaughterhouse and in food products; specifically, from minced meat preparations derived from animals originating from farms 1, 3, and 4, and from a cutting knife used during the slaughter of animals from farm 3. The genetic distance of approximately 1,645 alleles from the CC398 hub confirms that the ST39 cluster represents an independent lineage unrelated to the farm-circulating LA-MRSA population.

#### 3.2.3 ST15 isolate

A single *S. aureus* isolate of ST15 (BfR-ST-02950) was recovered from a cutting knife at the slaughterhouse during slaughtering of farm 4 animals. Its cgMLST profile was separated from all other isolates by approximately 1,739 alleles.

### 3.3 Virulence and antimicrobial resistance gene content

Screening of all 34 assemblies against the VFDB core dataset (ABRicate, 70% coverage/70% identity) detected 142 candidate virulence loci. ST398 isolates (*n* = 26) carried a mean of 108.3 loci (range: 107–110); all seven ST39 isolates carried an identical complement of 119 loci, and the single ST15 isolate carried 117 loci. Gene content differed categorically between the two MRSA lineages: the immune evasion cluster genes *scn* and *sak*, the *egc* enterotoxin cluster (*seg, selu, selo, seln*), and the enterotoxin-like gene *selv* were present in all seven ST39 isolates and absent from all 26 ST398 isolates (Fisher’s exact test, two-tailed, *p* < 0.001 for each gene; Table 4). The ST15 isolate carried a partial IEC profile (*chp*, *scn* positive; *sak* negative). The full 142-locus presence/absence matrix is provided in Supplementary Material S4.

**Table 4:** Key virulence genes differentiating the ST398 and ST39 MRSA lineages, and ST15 *S. aureus*.

Gene(s)	Function	ST398 (*n* = 26)	ST39 (*n* = 7)	ST15 (*n* = 1)	*p*
*scn*	IEC – complement inhibitor	0/26	7/7	1/1	<0.001
*sak*	IEC – staphylokinase	0/26	7/7	0/1	<0.001
*chp*	IEC – CHIPS	0/26	0/7	1/1	—
*seg, selu, selo, seln*	*egc* enterotoxin cluster	0/26	7/7	0/1	<0.001 (each)
*selv*	Enterotoxin-like V	0/26	7/7	0/1	<0.001
Total loci detected (mean)	—	108.3	119.0	117	—

*p*-values: Fisher’s exact test (two-tailed). Not calculated for *chp* (0/26 vs. 0/7 — absent in both lineages, no variation to test). No correction for multiple comparisons was applied, given the small, pre-specified gene panel identified from the cgMLST-based lineage grouping. The full 142-locus virulence gene presence/absence matrix is provided in Supplementary Material S4.

Isolates were additionally screened against reference sequences for the classical staphylococcal enterotoxin genes (*sea–see*, VF0020) and for the phage-encoded Panton-Valentine leukocidin genes (*lukS-PV*/*lukF-PV*, VF0018); none were detected in any of the 34 isolates.

AMR gene screening (AMRFinderPlus) of all 34 strains revealed a distinct, lineage-associated resistance gene complement that also mirrored the cgMLST-based clustering (Table 5). The 33 MRSA isolates carried *mecA*; the single ST15 *S. aureus* isolate lacked *mecA*, *mecR1*, and *mecI*, consistent with its designation as *mecA*-negative. *Tet(38)* and *blaI* were universally present (34/34), while *glpT_A100V*, *murA_D278E*, and *murA_E291D* (fosfomycin-associated) were near-universal (33/34), reflecting core genome background rather than lineage-specific acquisition. In contrast, several genes segregated sharply by lineage: the ST39 cluster (*n* = 7) uniquely carried *mecI/mecR1*, *aac(6′)-Ie/aph(2″)-Ia*, *bleO*, *qacC*, and *fosB* (all 7/7, absent from all 26 ST398 isolates; Fisher’s exact test, *p* < 0.001 for each), and was significantly enriched, though not fully exclusive, for *aadD1* (7/7 ST39 vs. 3/26 ST398; *p* < 0.001). Conversely, *tet(K)*, *tet(M)*, *dfrG*, *lnu(B)*, and *lsa(E)* were significantly more prevalent in the ST398 lineage (*n* = 26; 50–100% within-lineage prevalence) and absent from all ST39 strains (Fisher’s exact test, *p* < 0.001 for *tet(K)*, *tet(M)*, and *dfrG*; *p* = 0.027 for *lnu(B)* and *lsa(E)*). Given the small, pre-specified panel of lineage-differentiating genes tested (identified from the cgMLST/phylogenetic grouping rather than an unbiased genome-wide screen), no correction for multiple comparisons was applied. See Supplementary Material S4 for full details.

**Table 5:** Key antimicrobial resistance genes differentiating the ST398 and ST39 MRSA lineages.

Gene(s)	Resistance class	ST398 (*n* = 26)	ST39 (*n* = 7)	ST15 (*n* = 1)	*p*
*mecI*	Methicillin (*mec* regulator)	0/26	7/7	0/1	<0.001
*mecR1*	Methicillin (*mec* regulator)	0/26	7/7	0/1	<0.001
*aac(6′)-Ie/aph(2″)-Ia*	Aminoglycoside	0/26	7/7	0/1	<0.001
*aadD1*	Aminoglycoside	3/26	7/7	0/1	<0.001
*bleO*	Bleomycin	0/26	7/7	0/1	<0.001
*qacC*	Biocide/QAC efflux	0/26	7/7	0/1	<0.001
*fosB*	Fosfomycin	0/26	7/7	1/1	<0.001
*tet(K)*	Tetracycline	22/26	0/7	0/1	<0.001
*tet(M)*	Tetracycline	26/26	0/7	0/1	<0.001
*dfrG*	Trimethoprim	22/26	0/7	0/1	<0.001
*lnu(B)*	Lincosamide	13/26	0/7	0/1	0.027
*lsa(E)*	Lincosamide/pleuromutilin/streptogramin A	13/26	0/7	0/1	0.027

*p*-values: Fisher’s exact test (two-tailed). No correction for multiple comparisons was applied, given the small, pre-specified gene panel identified from the cgMLST-based lineage grouping. Genes universally present or absent across both lineages (e.g., *mecA*, *tet(38)*, *blaI*) are not shown; the full 36-locus AMR presence/absence matrix is provided in Supplementary Material S4.

## 4 Discussion

### 4.1 Farm MRSA prevalence and herd-level factors

This study provides cgMLST-confirmed evidence for the traceability of LA-MRSA (ST398) from colonized fattening pigs through slaughter and into fermented ready-to-eat meat products. To our knowledge, it represents one of the first studies worldwide to trace LA-MRSA across the complete food chain using WGS strongly indicating a farm-to-product transmission of LA-MRSA.

MRSA was detected on 75% (3/4) of farms included in the study, with all pooled animal nasal swabs positive. This is consistent with the high LA-MRSA burden documented in German pig farming and with broader European surveillance data. LA-MRSA have been found in pigs in Germany for more than 20 years (Meemken et al., 2010). Germany was also among the countries with the highest prevalence of MRSA (European Food Safety Authority, 2009, 2010; Alt et al., 2011). It has been pointed out that control of MRSA on pig farms is complex, especially due to the high risk of re-stocking farms with positive animals (Sawodny et al., 2025). Moreover, reduction of antimicrobial treatment of animals, one major driver of antimicrobial resistance at the One-Health interface (Fetsch and Johler, 2026), alone may not be sufficient. In the Netherlands, approximately 76% of Dutch pig farms remain MRSA-positive despite a 70% reduction in veterinary antimicrobial use since 2009, an observation that underscores the ecological entrenchment of ST398 in livestock (van Duijkeren et al., 2025). The absence of MRSA on farm 2 may be consistent with its lower stocking density and slaughter weight of older pigs (350 finishing places and a slaughter weight of 150 kg in farm 2 compared to 350 places and 118 kg in farm 1, 1,500 places and 118 kg in farm 3, and 4,500 places and 118 kg in farm 4), or factors previously associated with reduced LA-MRSA prevalence (Smith et al., 2009). However, sampling error may have contributed to this finding. The observation of multiple ST398 *spa* types (t034, t011, t2011, t1255) across farms and sampling stages, while consistent with co-circulating sub-lineages, may also reflect simultaneous carriage of genetically distinct MRSA clones within individual animals (Fetsch et al., 2016).

### 4.2 Farm-level pests as MRSA carriers

At the farm level, our cgMLST data provide molecular evidence that pest insects share genetically near-identical ST398 strains with the production animals with which they co-inhabit. These findings are consistent with an emerging body of evidence linking synanthropic flies to LA-MRSA carriage in pig production environments. Stelder et al. (2021) demonstrated that house flies (*Musca domestica*) and stable flies (*Stomoxys calcitrans*) on a Danish LA-MRSA-positive pig farm carried viable LA-MRSA on their body surfaces and that flies acquired LA-MRSA through passive environmental exposure without direct contact with pigs, suggesting airborne or surface-mediated uptake within the barn. More recently, a cross-sectional study across Austrian piglet-producing farms detected ST398 MRSA in 53.2% of house flies and 19.1% of stable flies on MRSA-positive holdings, providing direct evidence of routine fly carriage in pig farming environments (Hamar et al., 2025). Whether the fly isolates in our study were passive recipients of environmental ST398 contamination or active participants in within-farm transmission cannot be resolved from a cross-sectional design. Nevertheless, the genomic clustering of pest and pig isolates may indicate a shared, recent source and is consistent with pests functioning as biosecurity-relevant MRSA-carriers or even as components of the farm MRSA reservoir. These findings reinforce the importance of integrated pest management as part of LA-MRSA control on pig holdings.

### 4.3 Slaughterhouse as a potential transmission interface

The shared slaughterhouse seems to represent a structurally important interface in the transmission dynamics observed in this study. The molecular typing results at the slaughter level provide evidence of circulating clones in the slaughterhouse environment. The most parsimonious explanation is cross-contamination or strain exchange at the shared slaughterhouse, consistent with the capacity of lairage pens, personnel contact, and shared equipment to facilitate inter-farm MRSA transfer during slaughter and processing (Beneke et al., 2011; Bouchami et al., 2020). However, as no data on animal sourcing or breeding stock provenance were available for the participating farms, a common supplier origin contributing to this strain similarity cannot be excluded. The recovery of LA-MRSA from the band saw blade during slaughter further illustrates how slaughter and processing equipment can become contaminated with farm-specific MRSA strains and potentially serve as a vehicle for downstream cross-contamination, as previously described for stainless steel surfaces in abattoir environments (Beneke et al., 2011).

### 4.4 cgMLST-based farm-to-product traceability

Our findings at slaughter, processing, and the final meat product clearly provide cgMLST-level evidence of recent common ancestry of LA-MRSA ST398, thereby enabling the traceability of MRSA from farm to the final product. WGS-based typing is now recognized as the standard for farm-to-fork LA-MRSA surveillance, enabling both outbreak tracking and the detection of cross-transmission events that conventional typing cannot resolve (Fetsch and Johler, 2026). We argue for the added value of tracing individual strains, rather than reporting only prevalence rates, for understanding transmission dynamics along the food chain.

### 4.5 MRSA survival in processed meat products

Our results provide molecular evidence that LA-MRSA ST398 is not always eliminated during food processing and can persist in final pork products, including the two ready-to-eat fermented sausages tested in this study (*Frische Zwiebelmettwurst* and *cervelat-type* sausage) until the end of the shelf life, displaying a potential consumer health risk. Interestingly, MRSA was not detected in any of the *Hackepeter* samples, despite its detection in minced meat used for their preparation. This is in contrast with the detection of MRSA in minced meat from pork and beef in Austria (Petternel et al., 2014) and pork/beef mixed minced meat in the Czech Republic (Tegegne et al., 2021). The reason for the lack of MRSA detection was not determined in this study. General possible explanations, such as competitive exclusion by adventitious lactic acid bacteria, dilution of the contaminating inoculum below the detection limit of the qualitative enrichment method, or batch-size-dependent sampling variability therefore remain speculative. Effective elimination of MRSA in *Lyoner* by heat treatment (75 °C/45 min) is consistent with the near-zero prevalence of MRSA in cooked meat products reported in a global meta-analysis (Xing et al., 2025) and aligns with published thermal inactivation kinetics for *S. aureus* (Kennedy et al., 2005).

MRSA survival through the full seven-day shelf life of *Frische Zwiebelmettwurst* demonstrates that LA-MRSA ST398 can survive in a fermented ready-to-eat product marketed for direct consumption. MRSA has previously been shown to tolerate the mild pH and water-activity conditions characteristic of short-fermented raw sausages (Beneke et al., 2011). The fermentation parameters recorded in this study are consistent with this tolerance. The transient absence of MRSA at the 50-h sampling point, despite positive results before and after, most likely reflects fluctuations near the qualitative detection threshold of the enrichment method rather than genuine elimination and re-introduction. However, since no quantitative studies have been conducted, this is merely an unsubstantiated assumption. The single positive *cervelat-type* sample versus the consistently positive *Zwiebelmettwurst* results suggests that more intensive fermentation conditions, i.e., higher temperature (20 °C vs. 7 °C) over a longer period (96 h vs. 48 h), provide greater MRSA suppression. The combination of more pronounced acidification and lower water activity in sliceable raw sausages likely contributed to this outcome. pH/a_W_ trajectories (Supplementary Tables S1-7 and S1-8) show that inhibitory thresholds for *S. aureus* (a_W_ ≈ 0.83–0.86; pH ≈ 4.0–4.5) were not reached in either product. In *Frische Zwiebelmettwurst*, pH/a_W_ at end of shelf life ranged 5.15–5.47/0.972–0.985 across the three MRSA-positive farms — around one pH unit and over 0.10 a_W_ units above threshold. In *cervelat-type*, the more intensive regime achieved greater acidification and drying (pH 4.69–5.23; a_W_ 0.957–0.974 after fermentation) but still stayed above threshold; the single MRSA-positive *cervelat-type* sample (farm 4) had the mildest values recorded for that product. Nevertheless, it was unexpected to detect MRSA in the sausage after aging rather than during the manufacturing process.

### 4.6 Food safety implications and the slaughterhouse as a potential MRSA entry point

Although food is not considered a primary source of human MRSA acquisition, the presence of viable LA-MRSA in a fermented ready-to-eat product at the end of its shelf life has direct food safety relevance. Also, Lozano et al. (2020) emphasized that the presence of MRSA in food is a public health concern. The findings support continued inclusion of MRSA in coordinated zoonosis monitoring programs, as done at European level (EFSA and ECDC, 2025). We argue for the added value of tracing individual strains, rather than reporting only prevalence rates, for understanding transmission dynamics along the food chain. The findings of our study that human-associated MRSA strains can enter the meat chain at the slaughter stage and that raw material for multiple product types can become contaminated through used equipment further underline the importance of worker health and equipment hygiene as part of the prerequisite programs in pork processing (Ebert, 2018).

A distinct and food-safety-relevant finding was the exclusive detection of human-associated MRSA (ST39, *spa*-type, SCC*mec* type II) at the slaughter meat processing and food product stages, with no ST39 isolates recovered at any farm-level or slaughterhouse nasal swab sampling point. MRSA of ST39 and *spa*-type t007, carrying SCC*mec* II, have been detected earlier in pigs in China (Sapugahawatte et al., 2020). The identical cgMLST profiles of all seven ST39 isolates of our study, separated from the CC398 hub by approximately 1,645 alleles, confirm that this lineage entered the food chain independently of the farm-circulating LA-MRSA population and represents a single introduction event or a persistent, genetically stable source. This phylogenetic separation is corroborated at the gene-content level: all seven ST39 isolates carried the immune evasion cluster (*scn, sak*) and *egc* enterotoxin cluster, elements characteristically acquired by human-adapted *S. aureus* via the ΦSa3 prophage and typically absent from livestock-adapted CC398 (Kraushaar et al., 2017), whereas none of the 26 ST398 isolates carried these loci. Critically, ST39 strains were recovered from minced meat of all three farms whose pigs were slaughtered at the shared facility, and an ST39 isolate was simultaneously recovered from a cutting knife surface sampled during meat cutting. Since the knife was sampled during (not prior to) cutting operations, it is not possible to determine unambiguously whether the knife served as the source of meat contamination or was itself contaminated from the meat. Both scenarios implicate the slaughterhouse environment as a potential point of entry for this human-adapted lineage. Whole-genome AMR gene screening, however, reinforced this lineage separation: ST39 isolates (almost) uniformly carried *mecI*/*mecR1*, *aac(6′)-Ie/aph(2″)-Ia*, *aadD1*, *bleO*, and *qacC*, genes absent from all ST398 isolates and confirmed as statistically significant (*p* < 0.001 for each). ST39 isolates were also significantly enriched for *aad*D1 relative to ST398, although this gene was not fully exclusive to the lineage. Conversely, *tet*(K), *tet*(M), *dfr*G, *lnu*(B), and *lsa*(E) were significantly more prevalent in ST398 and absent from ST39. This largely lineage-associated resistance-gene profiles independently corroborates the cgMLST-based conclusion that ST39 is genetically and functionally distinct from the farm-circulating LA-MRSA population.

Nasal screening of all three project staff members at study commencement and completion returned negative MRSA results on both occasions. Therefore, it appears unlikely that human MRSA strains were introduced into the food chain by the project staff. However, the broader workforce of the shared slaughterhouse was not screened, and their MRSA carriage status remains unknown. Elevated nasal MRSA carriage rates among slaughterhouse workers in direct contact with live pigs have been well documented in European settings (Van Cleef et al., 2010), and the human-associated profile of the ST39 cluster is consistent with an occupational exposure route as a source of introduction. Shared equipment used across sequential farm batches within the same facility provides a plausible mechanistic pathway for the cross-farm distribution of such strains into the raw meat fraction.

### 4.7 Limitations

The study included only a small number of farms and animals (pigs and pests) and was conducted in a single geographic region and seasonal window; conditions at the single slaughterhouse may also not be representative. However, the intention of this study was not to provide prevalence data but to trace genetically identical MRSA throughout the pork farm-to-fork food chain and assess its survival in the end product. Still, individual-animal strain diversity may be underestimated: pooled nasal swabbing precludes individual traceability, and pigs can simultaneously carry multiple genetically distinct MRSA clones in the anterior nares (Fetsch et al., 2016), meaning that the isolate selected for sequencing from each pool may not capture the full strain repertoire present. Therefore, within-farm transmission can only be hypothesized and cannot be proven. Also, seasonal effects on MRSA occurrence were outside the scope of the present traceability study and are acknowledged as a limitation. The transient negative result in *Frische Zwiebelmettwurst* cannot be definitively interpreted without quantitative data. In addition, the definitive attribution of the human-associated strains to their source remains limited in the absence of screening results from slaughterhouse workers. Nasal swabs taken from animals at slaughter were collected ante-mortem for farms 3 and 4 but post-mortem for farm 1 due to logistical constraints, introducing a potential comparability limitation. Post-mortem swabbing may yield higher bacterial counts due to loss of mucosal defense mechanisms and altered sample accessibility, which could inflate apparent MRSA detection sensitivity for farm 1 relative to farms 3 and 4. However, since all slaughterhouse nasal swab pools were positive regardless of sampling method, this difference is unlikely to have affected the overall detection outcome in this study.

## 5 Conclusion

This study demonstrates that LA-MRSA of the ST398 lineage can be tracked along the production chain from fattening pig farms to ready-to-eat fermented meat products using cgMLST, with MRSA survival confirmed throughout the full shelf life of some products. The processing parameters applied to produce meat products were sometimes effective in eliminating MRSA, but MRSA was detectable during production and throughout the entire shelf life, particularly in short-ripened fermented sausages. The slaughterhouse may represent an entry point for human-associated MRSA into the food chain, underscoring slaughterhouse personnel and equipment hygiene as part of the prerequisite programs. At the farm level, flies should be recognized as potential vectors for MRSA transmission within and between farms. Overall, these findings support risk-based monitoring of MRSA in raw and minimally processed pork products and argue for whole-chain genomic surveillance to detect and interrupt farm-to-fork LA-MRSA transmission.

## Data Availability

The datasets presented in this study can be found in online repositories. The names of the repository/repositories and accession number(s) can be found at: https://www.ncbi.nlm.nih.gov/, BioProject PRJNA1477629.

## Footnotes

1
https://github.com/tseemann/abricate

## References

[ref1] Alt K. Fetsch A. Schroeter A. Guerra B. Hammerl J. A. Hertwig S. . (2011). Factors associated with the occurrence of MRSA CC398 in herds of fattening pigs in Germany. BMC Vet. Res. 7:69. doi: 10.1186/1746-6148-7-69, 22074403 PMC3260235

[ref2] Angen Ø. Nielsen M. W. Løfstrøm P. Larsen A. R. Hendriksen N. B. (2021). Airborne spread of methicillin resistant Staphylococcus aureus from a swine farm. Front. Vet. Sci. 8:644729. doi: 10.3389/fvets.2021.644729, 34150881 PMC8211894

[ref3] Beneke B. Klees S. Stührenberg B. Fetsch A. Kraushaar B. Tenhagen B.-A. (2011). Prevalence of methicillin-resistant Staphylococcus aureus in a fresh meat pork production chain. J. Food Prot. 74, 126–129. doi: 10.4315/0362-028X.JFP-10-250, 21219774

[ref4] Biguenet A. Bordy A. Atchon A. Hocquet D. Valot B. (2023). Introduction and benchmarking of pyMLST: open-source software for assessing bacterial clonality using core genome MLST. Microb Genom 9:001126. doi: 10.1099/mgen.0.001126, 37966168 PMC10711306

[ref5] Bisdorff B. Scholhölter J. L. Claußen K. Pulz M. Nowak D. Radon K. (2012). MRSA-ST398 in livestock farmers and neighbouring residents in a rural area in Germany. Epidemiol. Infect. 140, 1800–1808. doi: 10.1017/s0950268811002378, 22313681

[ref6] Bos M. E. Verstappen K. M. van Cleef B. A. Dohmen W. Dorado-García A. Graveland H. . (2016). Transmission through air as a possible route of exposure for MRSA. J. Expo. Sci. Environ. Epidemiol. 26, 263–269. doi: 10.1038/jes.2014.85 25515375

[ref7] Bouchami O. Fraqueza M. J. Faria N. A. Alves V. Lawal O. U. de Lencastre H. . (2020). Evidence for the dissemination to humans of methicillin-resistant Staphylococcus aureus ST398 through the pork production chain: a study in a Portuguese slaughterhouse. Microorganisms 8:1892. doi: 10.3390/microorganisms8121892, 33260448 PMC7759831

[ref8] Chen S. (2023). Ultrafast one-pass FASTQ data preprocessing, quality control, and deduplication using fastp. iMeta 2:e107. doi: 10.1002/imt2.107, 38868435 PMC10989850

[ref9] Chklovski A. Parks D. H. Woodcroft B. J. Tyson G. W. (2023). CheckM2: a rapid, scalable and accurate tool for assessing microbial genome quality using machine learning. Nat. Methods 20, 1203–1212. doi: 10.1038/s41592-023-01940-w, 37500759

[ref10] Dierikx C. M. Hengeveld P. D. Veldman K. T. de Haan A. van der Voorde S. Dop P. Y. . (2016). Ten years later: still a high prevalence of MRSA in slaughter pigs despite a significant reduction in antimicrobial usage in pigs the Netherlands. J. Antimicrob. Chemother. 71, 2414–2418. doi: 10.1093/jac/dkw190, 27261265

[ref11] Ebert M. (2018). “Chapter 11 - hygiene principles to avoid contamination/cross-contamination in the kitchen and during food processing,” in Staphylococcus Aureus, ed. Fetsch A. (Cambridge: Academic Press), 217–234.

[ref12] EFSA, and ECDC (2025). The European Union summary report on antimicrobial resistance in zoonotic and indicator bacteria from humans, animals and food in 2022–2023. EFSA J. 23:e9237. doi: 10.2903/j.efsa.2025.9237 PMC11880826 40046901

[ref13] EFSA Panel on Animal Health and Welfare Nielsen S. S. Bicout D. J. Calistri P. Canali E. Drewe J. A. . (2022). Assessment of listing and categorisation of animal diseases within the framework of the animal health law (regulation (EU) no 2016/429): antimicrobial-resistant Staphylococcus aureus in cattle and horses. EFSA J. 20:e07312. doi: 10.2903/j.efsa.2022.7312 PMC9087474 35582361

[ref14] European Food Safety Authority (2009). Analysis of the baseline survey on the prevalence of methicillin-resistant Staphylococcus aureus (MRSA) in holdings with breeding pigs, in the EU, 2008 - part a: MRSA prevalence estimates. EFSA J. 7:1376. doi: 10.2903/j.efsa.2009.1376 PMC13085198 42006962

[ref15] European Food Safety Authority (2010). Analysis of the baseline survey on the prevalence of methicillin-resistant Staphylococcus aureus (MRSA) in holdings with breeding pigs, in the EU, 2008 - part B: factors associated with MRSA contamination of holdings. EFSA J. 8:1597. doi: 10.2903/j.efsa.2010.1597, 42006962 PMC13085198

[ref16] Feldgarden M. Brover V. Gonzalez-Escalona N. Frye J. G. Haendiges J. Haft D. H. . (2021). AMRFinderPlus and the reference gene catalog facilitate examination of the genomic links among antimicrobial resistance, stress response, and virulence. Sci. Rep. 11:12728. doi: 10.1038/s41598-021-91456-0, 34135355 PMC8208984

[ref17] Fetsch A. Etter D. Johler S. (2021). Livestock-associated meticillin-resistant Staphylococcus aureus—current situation and impact from a one health perspective. Curr. Clin. Microbiol. Rep. 8, 103–113. doi: 10.1007/s40588-021-00170-y

[ref18] Fetsch A. Johler S. (2026). Staphylococcus aureus and S. argenteus in food systems: toxigenicity, resistance, and surveillance at the animal-human-environmental interface. Curr. Opin. Food Sci. 69:101415. doi: 10.1016/j.cofs.2026.101415

[ref19] Fetsch A. Kraushaar B. Käsbohrer A. Hammerl J. A. (2017). Turkey meat as source of CC9/CC398 methicillin-resistant Staphylococcus aureus in humans? Clin. Infect. Dis. 64, 102–103. doi: 10.1093/cid/ciw687, 27794020 PMC5159606

[ref20] Fetsch A. Roesler U. Kraushaar B. Friese A. (2016). Co-colonization and clonal diversity of methicillin-sensitive and methicillin-resistant Staphylococcus aureus in sows. Vet. Microbiol. 185, 7–14. doi: 10.1016/j.vetmic.2016.01.011, 26931385

[ref21] Fosheim G. E. Nicholson A. C. Albrecht V. S. Limbago B. M. (2011). Multiplex real-time PCR assay for detection of methicillin-resistant Staphylococcus aureus and associated toxin genes. J. Clin. Microbiol. 49, 3071–3073. doi: 10.1128/jcm.00795-11 PMC3147714 21697325

[ref22] Hamar F. Loncaric I. Bernreiter-Hofer T. Cabal Rosel A. Stöger A. Palle-Reisch M. . (2025). MRSA in pig farming: the emerging role of flies in antimicrobial resistance: a cross-sectional study. Porcine Health Manag 11:46. doi: 10.1186/s40813-025-00459-0, 41029438 PMC12486699

[ref23] Jolley K. A. Maiden M. C. (2010). BIGSdb: scalable analysis of bacterial genome variation at the population level. BMC Bioinformatics 11:595. doi: 10.1186/1471-2105-11-595, 21143983 PMC3004885

[ref24] Kennedy J. Blair I. S. McDowell D. A. Bolton D. J. (2005). An investigation of the thermal inactivation of Staphylococcus aureus and the potential for increased thermotolerance as a result of chilled storage. J. Appl. Microbiol. 99, 1229–1235. doi: 10.1111/j.1365-2672.2005.02697.x, 16238754

[ref25] Kilic A. Muldrew K. L. Tang Y. W. Basustaoglu A. C. (2010). Triplex real-time polymerase chain reaction assay for simultaneous detection of Staphylococcus aureus and coagulase-negative staphylococci and determination of methicillin resistance directly from positive blood culture bottles. Diagn. Microbiol. Infect. Dis. 66, 349–355. doi: 10.1016/j.diagmicrobio.2009.11.010, 20226325

[ref26] Kinross P. Petersen A. Skov R. Van Hauwermeiren E. Pantosti A. Laurent F. . (2017). Livestock-associated meticillin-resistant Staphylococcus aureus (MRSA) among human MRSA isolates, European Union/European economic area countries, 2013. Euro Surveill. 22:16-00696. doi: 10.2807/1560-7917.Es.2017.22.44.16-00696, 29113628 PMC5710135

[ref27] Kraushaar B. Fetsch A. (2014). First description of PVL-positive methicillin-resistant Staphylococcus aureus (MRSA) in wild boar meat. Int. J. Food Microbiol. 186, 68–73. doi: 10.1016/j.ijfoodmicro.2014.06.018 25016468

[ref28] Kraushaar B. Hammerl J. A. Kienöl M. Heinig M. L. Sperling N. Dinh Thanh M. . (2017). Acquisition of virulence factors in livestock-associated MRSA: lysogenic conversion of CC398 strains by virulence gene-containing phages. Sci. Rep. 7:2004. doi: 10.1038/s41598-017-02175-4, 28515479 PMC5435737

[ref29] Lozano C. Fernández-Fernández R. Ruiz-Ripa L. Gómez P. Zarazaga M. Torres C. (2020). Human mecC-carrying MRSA: clinical implications and risk factors. Microorganisms 8:1615. doi: 10.3390/microorganisms8101615, 33092294 PMC7589452

[ref30] Meemken D. Blaha T. Tegeler R. Tenhagen B. A. Guerra B. Hammerl J. A. . (2010). Livestock associated methicillin-resistant Staphylococcus aureus (LaMRSA) isolated from lesions of pigs at necropsy in Northwest Germany between 2004 and 2007. Zoonoses Public Health 57, e143–e148. doi: 10.1111/j.1863-2378.2009.01313.x, 20042059

[ref32] Petternel C. Galler H. Zarfel G. Luxner J. Haas D. Grisold A. J. . (2014). Isolation and characterization of multidrug-resistant bacteria from minced meat in Austria. Food Microbiol. 44, 41–46. doi: 10.1016/j.fm.2014.04.013, 25084643

[ref33] Prjibelski A. Antipov D. Meleshko D. Lapidus A. Korobeynikov A. (2020). Using SPAdes De novo assembler. Curr. Protoc. Bioinformatics 70:e102. doi: 10.1002/cpbi.102, 32559359

[ref34] Sapugahawatte D. N. Li C. Yeoh Y. K. Dharmaratne P. Zhu C. Ip M. (2020). Swine methicillin-resistant Staphylococcus aureus carrying toxic-shock syndrome toxin gene in Hong Kong, China. Emerg. Microbes Infect. 9, 1534–1536. doi: 10.1080/22221751.2020.1785335 PMC7473289 32573344

[ref35] Sawodny S. Käsbohrer A. Bröker L. Firth C. Marschik T. (2025). Intervention strategies for methicillin-resistant Staphylococcus aureus control in pig farming: a comprehensive review. Porcine Health Management 11:17. doi: 10.1186/s40813-025-00435-8, 40181479 PMC11969979

[ref36] Shen W. Sipos B. Zhao L. (2024). SeqKit2: a Swiss army knife for sequence and alignment processing. iMeta 3:e191. doi: 10.1002/imt2.191, 38898985 PMC11183193

[ref37] Smith T. C. Male M. J. Harper A. L. Kroeger J. S. Tinkler G. P. Moritz E. D. . (2009). Methicillin-resistant Staphylococcus aureus (MRSA) strain ST398 is present in midwestern U.S. swine and swine workers. PLoS One 4:e4258. doi: 10.1371/journal.pone.0004258, 19145257 PMC2626282

[ref38] Stelder J. J. Kjær L. J. Jensen L. B. Boklund A. E. Denwood M. Carlsen M. . (2021). Livestock-associated MRSA survival on house flies (Musca domestica) and stable flies (Stomoxys calcitrans) after removal from a Danish pig farm. Sci. Rep. 11:3527. doi: 10.1038/s41598-021-83228-7, 33574465 PMC7878913

[ref39] Tegegne H. A. Koláčková I. Florianová M. Gelbíčová T. Madec J. Y. Haenni M. . (2021). Detection and molecular characterisation of methicillin-resistant Staphylococcus aureus isolated from raw meat in the retail market. J Glob Antimicrob Resist 26, 233–238. doi: 10.1016/j.jgar.2021.06.012, 34271219

[ref40] Van Cleef B. A. Broens E. M. Voss A. Huijsdens X. W. Züchner L. Van Benthem B. H. . (2010). High prevalence of nasal MRSA carriage in slaughterhouse workers in contact with live pigs in the Netherlands. Epidemiol. Infect. 138, 756–763. doi: 10.1017/s0950268810000245 20141647

[ref41] van Duijkeren E. Brouwer M. S. M. Wullings B. Rapallini M. Wit B. Cuperus T. . (2025). Methicillin-resistant Staphylococcus aureus GG0398 on livestock farms and meat in the Netherlands. J. Glob. Antimicrob. Resist. 43, 79–85. doi: 10.1016/j.jgar.2025.04.007 40258490

[ref42] Wang W. Baker M. Hu Y. Xu J. Yang D. Maciel-Guerra A. . (2021). Whole-genome sequencing and machine learning analysis of Staphylococcus aureus from multiple heterogeneous sources in China reveals common genetic traits of antimicrobial resistance. mSystems 6:e0118520. doi: 10.1128/msystems.01185-20 PMC8579812 34100643

[ref43] Xing L. Cheng M. Wang S. Jiang J. Li T. Zhang X. . (2025). Methicillin-resistant Staphylococcus aureus contamination in meat and meat products: a systematic review and meta-analysis. Front. Microbiol. 16:1636622. doi: 10.3389/fmicb.2025.1636622, 40735619 PMC12303875

[ref44] Zhou S. Liu B. Zheng D. Chen L. Yang J. (2025). VFDB 2025: an integrated resource for exploring anti-virulence compounds. Nucleic Acids Res. 53, D871–d877. doi: 10.1093/nar/gkae968, 39470738 PMC11701737

