# Supplementary material for: Livestock-associated methicillin-resistant Staphylococcus aureus along the pork food chain: a study of four commercial pig fattening farms in north-east Germany and survival in processed meat products
Source: Front Microbiol. 2026 Oct 27;17:1928317. doi: 10.3389/fmicb.2026.1928317 (PMC13748208; doi:10.3389/fmicb.2026.1928317)
Supplement: Supplementary file 1 [file Table_1.docx]

**Supplementary Material S1**

**Livestock-associated methicillin-resistant *Staphylococcus aureus* along the pork food chain: A study of four commercial pig fattening farms in North-East Germany and survival in processed meat products**

Marco Ebert^1*^, Sandra Rose^1,2^, Michael Biggel^3^, Alexandra Fetsch^4,5^

^1^ Department of Agriculture and Food Sciences, Neubrandenburg University of Applied Sciences (HS NB), Neubrandenburg, Germany

^2^ Faculty of Agricultural and Environmental Sciences, University of Rostock, Rostock, Germany

^3^ Institute of Food Safety and Hygiene, University of Zurich (UZH), Zurich, Switzerland

^4^ Competence Centre for Food Safety, Chair of Food Microbiology, Faculty of Veterinary Medicine, Ludwig-Maximilians-Universität München (LMU Munich), Munich, Germany

^5^ Department Biological Safety, German Federal Institute for Risk Assessment (BfR), Berlin, Germany

*Corresponding Author:

Marco Ebert

Neubrandenburg University of Applied Sciences

Email: [ebert@hs-nb.de](mailto:ebert@hs-nb.de)

**Table S1-1: Meat samples taken from the slaughtered animals**

| **Part of carcasses** | **Grade classification according to the GEHA system of the German Butchers' Association** |
| --- | --- |
| lean cuts | S II |
| back fat | S VIII |
| belly meat | S IV |
| cheek meat | S VI |

**Table S1-2: Ingredients for the production of seasoned minced meat (Hackepeter)**

| **Mass (kg)** | **Mass (%)** | **Ingredient** |
| --- | --- | --- |
| 0,325 | 54,08 | lean cuts (Slaughterhouse) |
| 0,225 | 37,44 | belly meat (Slaughterhouse) |
| 0,050 | 8,32 | Onions (Heilmann AG, München, Germany) |
| 0,001 | 0,16 | black, ground pepper (Hallesche Essig- und Senffabrik GmbH, Bad Dürrenberg, Germany) |
| 0,601 | 100 | Total |

**Table S1-3: Ingredients for the production of the spreadable raw sausages (Frische Zwiebelmettwurst)**

| **Mass (kg)** | **Mass (%)** | **Ingredient** |
| --- | --- | --- |
| 0,1985 | 39,54 | lean cuts (Slaughterhouse) |
| 0,2360 | 47,01 | belly meat (Slaughterhouse) |
| 0,0480 | 9,96 | Onions (Heilmann AG, München, Germany) |
| 0,0010 | 0,2 | black, ground pepper (Hallesche Essig- und Senffabrik GmbH, Bad Dürrenberg, Germany) |
| 0,0010 | 0,2 | rubbed marjoram (Hallesche Essig- und Senffabrik GmbH, Bad Dürrenberg, Germany) |
| 0,0100 | 1,99 | Nitrite curing salt (0,4 - 0,5 % sodium nitrite, Südwestdeutsche Salzwerke AG, Heilbronn, Germany) |
| 0,0050 | 1 | Hela2000 raw sausage ripening agent for coarse-textured Mettwurst O. G. (Ingredients: Spices, Acidity regulator [E575 glucono-delta-lactone 25%], dextrose, lactose, antioxidants [E301 sodium L-ascorbate, E300 L-ascorbic acid]), Hela Gewürzwerk Hermann Laue GmbH, Ahrensburg, Germany |
| 0,0005 | 0,1 | “Germet 2000” starter cultures for firm raw sausage (Ingredients: Dextrose, freeze-dried starter cultures [*Staphylococcus carnosus* and *Lactobacillus curvatus*]), Hela Gewürzwerk Hermann Laue GmbH, Ahrensburg, Germany |
| 0,5000 | 100 | Total |

**Table S1-4: Ingredients for the production of the sliceable raw sausage (cervelat-type)**

| **Mass (kg)** | **Mass (%)** | **Ingredient** |
| --- | --- | --- |
| 3,500 | 67,63 | lean cuts (Slaughterhouse) |
| 1,500 | 28,99 | back fat (Slaughterhouse) |
| 0,095 | 1,84 | Nitrite curing salt (0,4 - 0,5 % sodium nitrite, Südwestdeutsche Salzwerke AG, Heilbronn, Germany) |
| 0,075 | 1,45 | Purpurit spice blend for dry-cured sausage, Thuringian Zervelat sausage (Ingredients: Spices [including mustard flour], dextrose [33%], antioxidant [E301 sodium L-ascorbate, E300 L-ascorbic acid]), Hela Gewürzwerk Hermann Laue GmbH, Ahrensburg, Germany |
| 0,005 | 0,097 | “Germet 2000” starter cultures for firm raw sausage (Ingredients: Dextrose, freeze-dried starter cultures [*Staphylococcus carnosus* and *Lactobacillus curvatus*]), Hela Gewürzwerk Hermann Laue GmbH, Ahrensburg, Germany |
| 5,175 | 100 | Total |

**Table S1-5: Ingredients for the production of the cooked sausage (Lyoner)**

| **Mass (kg)** | **Mass (%)** | **Ingredient** |
| --- | --- | --- |
| 2,44 | 45,66 | lean cuts (Slaughterhouse) |
| 1,44 | 26,97 | back fat (Slaughterhouse) |
| 0,25 | 4,67 | cheek meat (Slaughterhouse) |
| 1,00 | 18,69 | crushed ice |
| 0,01 | 0,19 | Onions (Heilmann AG, München, Germany) |
| 0,04 | 0,65 | Helabin “Ultimal” Cutter Aid – A cutter aid for use with cooked sausage combined with reddening agents and flavor enhancers (Ingredients: stabilizer [E450 diphosphates*], acidity regulator [E575 glucono-delta-lactone], dextrose, antioxidants [E301 sodium L-ascorbate, E300 L-ascorbic acid], flavor enhancer [E621 monosodium glutamate], spices (including celery), acidifier: [E330 citric acid], flavoring *for food), Hela Gewürzwerk Hermann Laue GmbH, Ahrensburg, Germany |
| 0,08 | 1,5 | Spice mix for cooked sausages (Ingredients: spices and herbs [contains mustard flour, celery], dextrose, table salt, flavorings), Hela Gewürzwerk Hermann Laue GmbH, Ahrensburg, Germany |
| 0,09 | 1,68 | Nitrite curing salt (0,4 - 0,5 % sodium nitrite, Südwestdeutsche Salzwerke AG, Heilbronn, Germany) |
| 5,35 | 100 | Total |

**Table S1-6: Arithmetic means of the pH value (n = 3) and the a_W_ value (n = 2) for the manufacture of seasoned raw minced meat (Hackepeter) made from the meat of pigs from the 3 MRSA-positive farms (Farm 1, 3 and 4), at various production steps**

| **production steps** | **Farm 1** | | **Farm 3** | | **Farm 4** | |
| --- | --- | --- | --- | --- | --- | --- |
|  | **pH** | **a_W_** | **pH** | **a_W_** | **pH** | **a_W_** |
| **raw minced meat** | 5,63 | 0,992 | 5,51 | 0,996 | 5,72 | 0,993 |
| **after production** | 5,59 | 0,986 | 5,68 | 0,997 | 5,71 | 0,993 |
| **after 24 h** | 5,72 | 0,993 | 5,54 | 0,994 | 5,30 | 0,994 |

**Tabel S1-7: Arithmetic means of the pH value (n = 3) and the a_W_ value (n = 2) for the manufacture of spreadable raw sausage (Frische Zwiebelmettwurst) made from the meat of pigs from the 3 MRSA-positive farms (Farm 1, 3 and 4), at various production steps**

| **production steps** | **Farm 1** | | **Farm 3** | | **Farm 4** | |
| --- | --- | --- | --- | --- | --- | --- |
|  | **pH** | **a_W_** | **pH** | **a_W_** | **pH** | **a_W_** |
| **raw minced meat** | 5,80 | 0,998 | 5,75 | 0,993 | 5,73 | 0,991 |
| **after filling** | 5,57 | 0,984 | 5,63 | 0,982 | 5,44 | 0,980 |
| **after ripening (24 h)** | 5,49 | 0,974 | 5,61 | 0,982 | 5,36 | 0,985 |
| **after storage (7 d)** | 5,28 | 0,972 | 5,15 | 0,980 | 5,47 | 0,985 |

**Table S1-8: Arithmetic means of the pH value (n = 3) and the a_W_ value (n = 2) for the manufacture of sliceable raw sausage (cervelat-type) made from the meat of pigs from the 3 MRSA-positive farms (Farm 1, 3 and 4), at various production steps**

| **production steps** | **Farm 1** | | **Farm 3** | | **Farm 4** | |
| --- | --- | --- | --- | --- | --- | --- |
|  | **pH** | **a_W_** | **pH** | **a_W_** | **pH** | **a_W_** |
| **raw minced meat** | 5,84 | 0,993 | 5,88 | 0,997 | 5,56 | 0,987 |
| **after filling** | 5,45 | 0,976 | 5,38 | 0,978 | 5,40 | 0,983 |
| **after ripening (4 d)** | 4,80 | 0,970 | 5,11 | 0,957 | 5,23 | 0,974 |
| **after storage (3 d)** | 4,69 | 0,969 | 5,06 | 0,976 | 5,03 | 0,977 |

**Table S1-9: Arithmetic means of the pH value (n = 3) and the a_W_ value (n = 2) for the manufacture of cooked sausages (Lyoner) made from the meat of pigs from the 3 MRSA-positive farms (Farm 1, 3 and 4), at various production steps**

| **production steps** | **Farm 1** | | **Farm 3** | | **Farm 4** | |
| --- | --- | --- | --- | --- | --- | --- |
|  | **pH** | **a_W_** | **pH** | **a_W_** | **pH** | **a_W_** |
| **raw minced meat** | 5,81 | 0,998 | 5,84 | 0,992 | 5,83 | 0,996 |
| **after cooking** | 5,81 | 0,974 | 5,78 | 0,978 | 5,71 | 0,987 |
| **after storage (7 d)** | 5,74 | 0,982 | 5,89 | 0,981 | 5,86 | 0,979 |
